# Balneotherapy and Spa Rehabilitation in Patients with Hematological Malignancies: Current Evidence, Clinical Safety, and a Proposed Qualification Framework—A Critical Narrative Review

**DOI:** 10.3390/jcm15218289

**Published:** 2026-10-24

**Authors:** Amanda Maria Kostro, Zofia Dzięcioł-Anikiej, Marta Sobas, Maria Czyżewska, Jacek Chojnowski, Agnieszka Staniak, Dominik Romiński, Adam Kopciewski, Mariusz Baumgart

**Affiliations:** 1 Department of Personalized Physiotherapy of the Musculoskeletal System, Faculty of Health Sciences, Medical University of Bialystok, M. Skłodowskiej-Curie 7a Street, 15-096 Bialystok, Poland; adam.kopciewski@umb.edu.pl; 2 Physical Activity Research Laboratory, Clinical Research Center, Medical University of Bialystok, M. Skłodowskiej-Curie Street 24a, 15-276 Bialystok, Poland; 3 Department of Hematology, Faculty of Medicine, Collegium Medicum in Bydgoszcz, Nicolaus Copernicus University in Torun, Ujejskiego Street 75, 85-168 Bydgoszcz, Poland; marta.sobas@cm.umk.pl; 4 Hematology Department, The Ludwik Rydygier Provincial Hospital in Torun, St. Joseph Street 53–59, 87-100 Torun, Poland; marysky@wp.pl; 5 Ciechocinek Health Resort, Piłsudskiego Street 3, 87-720 Ciechocinek, Poland; jacchojnowski@gmail.com; 6 InesFizjo Physiotherapy, Kminkowa 2/4, 80-177 Gdansk, Poland; agnieszka.as.staniak@gmail.com; 7 Direction Health Association, 24 Mickiewicza St., 87-100 Torun, Poland; kontakt@kierunekzdrowie.org; 8 oncoAlliance, Wawelska Street 15b/3, 04-881 Warsaw, Poland; 9 Initiative for Hematological Rehabilitation, 24 Mickiewicza St., 87-100 Torun, Poland; 10 Department of Anatomy, Faculty of Medicine, Collegium Medicum in Bydgoszcz, Nicolaus Copernicus University in Torun, Łukasiewicza Street 1, 85-821 Bydgoszcz, Poland; mariusz.baumgart@cm.umk.pl

**Keywords:** hematological malignancies, balneotherapy, spa therapy, hydrotherapy, rehabilitation, exercise, clinical safety, hematopoietic stem cell transplantation, patient selection

## Abstract

**Background:** Hematological malignancies and their treatment frequently cause fatigue, reduced physical capacity, neuropathy, pain and impaired quality of life. Exercise-based rehabilitation is increasingly recognized as part of the care of patients with hematological malignancies, whereas the role and safety of spa-based interventions, such as balneotherapy, hydrotherapy and peloidotherapy, remain uncertain. **Methods:** A critical narrative review of PubMed was performed. Evidence obtained in patients with hematological malignancies (direct evidence) was distinguished from evidence obtained in other oncological and non-oncological populations (indirect evidence), and each recommendation was assigned an evidence-certainty label (A–D). **Results:** No clinical studies evaluating balneotherapy or spa therapy in hematological malignancies were identified. Evidence supports exercise-based and functional rehabilitation for improving physical capacity, fatigue and quality of life, including in selected patients with cytopenias. Evidence for spa-specific procedures is indirect, and the laboratory thresholds used in practice derive from exercise studies and expert opinion rather than from validation for spa-specific procedures. **Conclusions:** The benefits documented in this population relate to rehabilitation and exercise, which can also be delivered in a spa setting; spa-specific procedures should be regarded as optional adjuncts requiring procedure-specific qualification. As a hypothesis for prospective evaluation, we also outline a framework in which the time-based eligibility criterion used in Poland is supplemented by structured clinical assessment.

## 1. Introduction

Treatment outcomes in many hematological malignancies have improved, and a growing number of patients live for years with the consequences of the disease and its treatment. Chemotherapy, hematopoietic stem cell transplantation and modern immunological therapies may be associated with cytopenias, infections, bleeding, neuropathy, fatigue and decreased physical performance. Rehabilitation and regular physical activity are considered important elements of comprehensive care in hematological malignancies, provided that individual qualification and monitoring are performed [1,2,3,4,5,6,7].

In several Central European countries, including Poland, rehabilitation is also delivered in spa (health resort) settings, where programs combine therapeutic exercise with balneological procedures (mineral baths, hydrotherapy, peloidotherapy), thermal and aerosol procedures and physical therapy modalities. Data on balneotherapy and spa treatment in hematological malignancies are scarce, and no clinical trials have directly evaluated these interventions in this population. Studies in other oncological populations and in chronic diseases, including rheumatic diseases, suggest possible improvements in quality of life, fatigue, pain and physical fitness, as well as changes in selected inflammatory and immunological markers [8,9,10,11,12,13,14,15,16,17,18,19], but their applicability to hematological patients is uncertain.

Several specific clinical uncertainties, therefore, arise: (i) whether spa rehabilitation offers any benefit beyond exercise-based rehabilitation delivered in other settings; (ii) which spa-specific procedures carry specific risks for hematological patients—infectious (water and aerosol exposure), hemorrhagic (manual and mechanical stimuli) or thermal and hemodynamic (heat exposure); (iii) which clinical and laboratory criteria should guide qualification, and from what evidence these criteria derive; and (iv) whether fixed waiting periods after anticancer treatment, such as those used in Poland, reflect the individual risk of the patient.

Spa treatment should not be considered anticancer therapy but, at most, a complement to rehabilitation in appropriately qualified, clinically stable patients. The core of any program should remain therapeutic exercise, functional training and individually prescribed physical activity, whereas spa-specific procedures and physical therapy modalities should be selected according to the current risk profile, type of treatment, complications and the feasibility of performing a given procedure safely.

The aim of this critical narrative review is to evaluate the available evidence on spa rehabilitation, balneotherapy and selected physical therapy modalities in patients with hematological malignancies, with explicit separation of direct and indirect evidence and particular emphasis on clinical safety. We also present a proposed framework for individual qualification and identify gaps requiring further research.

## 2. Hematological Malignancies: Clinical Context

### 2.1. Disease Spectrum and Treatment-Related Complications

Hematological malignancies comprise myeloid and lymphoid neoplasms with acute and chronic courses [20]. In acute leukemias, including acute myeloid leukemia (AML) and acute lymphoblastic leukemia (ALL), the proliferation of blasts displaces normal hematopoiesis, leading to anemia, thrombocytopenia and neutropenia [21,22,23,24]. Chronic myeloproliferative neoplasms include chronic myeloid leukemia (CML) and the Philadelphia chromosome-negative myeloproliferative neoplasms (MPN Ph−): essential thrombocythemia (ET), polycythemia vera (PV) and myelofibrosis (MF). Chronic lymphoid neoplasms include chronic lymphocytic leukemia (CLL) and lymphomas, and multiple myeloma is a distinct plasma cell neoplasm [25,26,27,28,29,30]. Treatment includes chemotherapy, monoclonal antibodies, targeted therapies, autologous and allogeneic hematopoietic stem cell transplantation (autoHSCT and alloHSCT), chimeric antigen receptor T-cell (CAR-T) therapy and, in selected situations, radiotherapy [31,32]. The treatment-related complications most relevant to rehabilitation are cytopenias, infections, bleeding, thrombosis and peripheral neuropathy [1,33,34,35,36,37]. The intensity of rehabilitation must, therefore, be adjusted to the type of disease, treatment stage, blood counts, infectious, hemorrhagic and thrombotic risk and general condition. Cytopenia itself does not necessarily preclude rehabilitation but requires appropriate supervision and modification of the exercise load [1,2,38].

### 2.2. Epidemiology and Survival

Survival in many hematological malignancies has improved over recent decades [39]. In some chronic diseases, survival now extends well beyond 10 years: in CML treated with tyrosine kinase inhibitors, life expectancy approaches that of the general population [40], and in a large molecularly annotated cohort, median survival was 19.8 years in ET and 13.5 years in PV [41]. Consequently, an increasing number of patients live for many years with the sequelae of the disease and its treatment.

In Poland, 11,434 new cases of malignant neoplasms of lymphoid, hematopoietic and related tissue (ICD-10 C81–C96) were registered in 2023, accounting for approximately 6% of the 192,922 malignant neoplasms registered that year [42]. The most frequent included lymphoid leukemia, non-follicular lymphoma and multiple myeloma (Table 1). Incidence is highest in older age groups, and a further increase in the number of cases is expected [42,43]. Analyses based on National Health Fund data indicate that the number of people living with these diagnoses is considerably higher than the annual number of new cases [44].

## 3. Methods

### 3.1. Design and Scope

This study is a critical narrative review. Its reporting was guided by the Scale for the Assessment of Narrative Review Articles (SANRA) [45]. A narrative rather than a systematic design was chosen because the research question spans heterogeneous interventions, populations and types of sources (clinical studies, guidelines and regulatory documents), and because a preliminary search indicated an absence of clinical studies of spa-based interventions in hematological malignancies.

This review covers spa rehabilitation as it is delivered in practice. In Poland and other Central European countries, a spa stay typically combines therapeutic exercise with balneological procedures (mineral baths, hydrotherapy, peloidotherapy), thermal and aerosol procedures and physical therapy modalities, such as electrotherapy, therapeutic ultrasound, photobiomodulation (laser therapy), extracorporeal shock wave therapy and massage. Qualification for spa rehabilitation, therefore, implies qualification for these procedures, each of which carries a distinct risk profile in hematological patients (infectious, hemorrhagic, thermal or local tissue risk). For this reason, selected physical therapy modalities were included, although balneotherapy and spa rehabilitation remain the primary focus of this review. For consistency, the following terms are used throughout: “spa rehabilitation” denotes the overall rehabilitation program delivered in a spa (health resort) setting; “spa-specific procedures” denotes balneological procedures (mineral baths, hydrotherapy and peloidotherapy) together with thermal and aerosol procedures; “water-based procedures” denotes hydrotherapy, aquatic exercise, mineral baths and pools; and “physical therapy modalities” denotes electrotherapy, therapeutic ultrasound, photobiomodulation, extracorporeal shock wave therapy and massage.

### 3.2. Literature Search

A structured literature search was performed in PubMed/MEDLINE. The primary search covered publications from January 2000 to October 2026, with the final search performed on 4 October 2026. Because the review was designed to assess contemporary clinical practice and current rehabilitation concepts, the main evidence synthesis focused on the 2000–2026 period. The search strategy used combinations of MeSH terms and free-text terms covering three principal concepts: (1) hematological malignancies and hematological rehabilitation; (2) balneotherapy, spa-based and related rehabilitation interventions; and (3) safety and clinically relevant outcomes.

The population-related search terms included the following:

(“Hematologic Neoplasms”[MeSH Terms] OR “Leukemia”[MeSH Terms] OR “Lymphoma”[MeSH Terms] OR “Multiple Myeloma”[MeSH Terms] OR leukemia*[Title/Abstract] OR lymphoma*[Title/Abstract] OR myeloma*[Title/Abstract] OR “hematologic malignan*”[Title/Abstract] OR “haematologic malignan*”[Title/Abstract] OR “hematologic disease*”[Title/Abstract] OR “haematologic disease*”[Title/Abstract] OR “Hematopoietic Stem Cell Transplantation”[MeSH Terms] OR “CAR-T”[Title/Abstract])

The intervention-related terms included the following:

(“Balneology”[MeSH Terms] OR “Hydrotherapy”[MeSH Terms] OR “Mud Therapy”[MeSH Terms] OR balneotherap*[Title/Abstract] OR hydrotherap*[Title/Abstract] OR “spa therapy”[Title/Abstract] OR “spa treatment”[Title/Abstract] OR pelotherap*[Title/Abstract] OR peloid*[Title/Abstract] OR “mud bath*”[Title/Abstract] OR “mud therap*”[Title/Abstract] OR “mineral bath*”[Title/Abstract] OR “thermal bath*”[Title/Abstract] OR “aquatic exercise”[Title/Abstract] OR “Physical Therapy Modalities”[MeSH Terms] OR “Rehabilitation”[MeSH Terms] OR “Exercise”[MeSH Terms])

Additional searches combined the intervention terms with safety- and outcome-related terms, including adverse events, infection, bleeding, thrombocytopenia, neutropenia, quality of life, fatigue, physical function, inflammatory markers, immune function, hemodynamic parameters and microcirculation.

To identify indirect evidence relevant to spa rehabilitation, additional searches combined the intervention terms with broader oncological and non-oncological populations, including other malignancies and chronic musculoskeletal diseases. Particular attention was paid to studies reporting biological, inflammatory, immunological, hematological, cardiovascular or functional outcomes that could inform the interpretation of spa-specific procedures in hematological patients.

Reference lists of relevant publications, systematic reviews, guidelines and consensus documents were additionally screened manually. Relevant recommendations and regulatory information were obtained from the websites of the organizations responsible for issuing the respective guidelines, consensus statements, registry reports and Polish legal or regulatory documents.

The search was not intended to constitute a systematic review search with exhaustive retrieval and quantitative study selection. Instead, it was designed to identify the principal body of evidence necessary to critically evaluate the clinical rationale, potential benefits, safety considerations and qualification criteria for spa rehabilitation in hematological malignancies. The search was restricted to publications in English and Polish. Accordingly, the numbers of records retrieved, screened and excluded are not reported, and no PRISMA flow diagram is provided.

### 3.3. Eligibility of Sources

Sources were considered eligible when they contributed relevant evidence to at least one of the following domains:

1. Rehabilitation, exercise or functional recovery in patients with hematological malignancies;
2. Balneotherapy, hydrotherapy, aquatic exercise, peloidotherapy, thermal procedures or other spa-based interventions in patients with cancer;
3. Physiological, inflammatory, immunological, hematological or cardiovascular effects of balneotherapy or related procedures in non-hematological populations;
4. Safety, contraindications, precautions or qualification criteria relevant to physical rehabilitation or spa-specific procedures in patients with cancer or hematological malignancies;
5. Clinical guidelines, consensus statements or regulatory documents relevant to rehabilitation, infection prevention, treatment-related complications or eligibility for health-resort treatment.

Randomized controlled trials, non-randomized and observational studies, case series, systematic reviews, meta-analyses and scoping reviews were considered. Narrative reviews were used for background information and mechanistic context. Clinical guidelines and consensus statements were used to inform safety and clinical qualification, while official epidemiological and regulatory documents were used for disease burden and the Polish regulatory context.

Animal and in vitro studies were not considered clinical evidence. Studies in healthy volunteers were used only when they provided relevant physiological or mechanistic information and were explicitly classified as indirect evidence. Non-institutional commercial web content was excluded.

### 3.4. Selection and Data Extraction

Records were screened by title and abstract and then in full text by two authors (A.M.K. and Z.D.-A.) independently; disagreements were resolved by discussion or, when necessary, by a third author (M.B.).

Because this was a critical narrative review rather than a systematic review, the selection process was focused on identifying the most relevant and clinically informative evidence rather than on estimating a pooled treatment effect.

For each included source, the following information was considered: study design, population and underlying disease, sample size, intervention and setting, duration and frequency of treatment, comparator where applicable, clinical and functional outcomes, hematological or biological outcomes, reported adverse events, and the relevance of the findings to patients with hematological malignancies.

Each source was subsequently classified according to the degree to which its findings could be directly applied to hematological rehabilitation or had to be extrapolated from another population, intervention or clinical context.

### 3.5. Classification of Evidence and Synthesis

Because this review combines evidence obtained in different populations, each recommendation and each entry in Table 2 was assigned an evidence-certainty label based on the concept of indirectness used in the GRADE approach. The evidence-certainty labels for the recommendations and entries presented in Table 3, Table 4, Table 5, Table 6, Table 7 and Table 8 are summarized in Table 2 [46]. Labels B–D indicate that a recommendation is extrapolated, and label D also covers recommendations representing the authors’ consensus. No formal risk-of-bias assessment of individual studies was performed, given the heterogeneity of study designs and the narrative scope of the review; this limitation is discussed in Section 11. Owing to the heterogeneity of populations, interventions and endpoints and the lack of head-to-head studies, no quantitative synthesis was performed. The results of indirect studies were interpreted with caution and were not treated as evidence of the effectiveness of balneotherapy in hematological malignancies.

## 4. Evidence for Rehabilitation in Hematological Malignancies

### 4.1. Effectiveness and Safety

Exercise-based and functional rehabilitation is the best-documented intervention in hematological malignancies (label A). In a retrospective analysis of inpatient hematology and oncology rehabilitation, published as a conference abstract, significant functional improvement and discharge home were reported in more than 70% of patients, including those with advanced disease [47]. Patients with acute leukemia undergoing intensive treatment, including alloHSCT, experience deterioration of physical, psychological and social functioning, which justifies early, individualized rehabilitation [48]. In patients with lymphoma and other hematological diseases with low blood counts, rehabilitation performed with the physician’s consent and appropriate monitoring was not associated with a significant increase in adverse events, and in some analyses, it was associated with shorter hospitalization and maintenance of functional capacity [4,5]. A review of studies in thrombocytopenic patients concluded that physical exercise is safe and feasible under appropriate supervision [7], and a recent review confirmed the importance of physical activity across hematological malignancies, with the need for individualized programs [6].

### 4.2. Timing and Setting

Rehabilitation should start as early as possible, including during inpatient and outpatient treatment. In the acute phase, the priorities are maintenance of function and prevention of immobility, using breathing exercises, bedside mobilization and training in activities of daily living (ADL). During convalescence, activity should be increased gradually, and in the stable phase of the disease, more comprehensive endurance and strength programs can be implemented [3,4,5,6,7,49]. A spa stay, where available, usually falls into this last phase and should be regarded as one possible setting for continued rehabilitation rather than as a separate treatment.

### 4.3. Rehabilitation During and After HSCT and CAR-T

AutoHSCT, alloHSCT and CAR-T therapy may lead to sarcopenia, cachexia and reduced exercise capacity and may increase cardiovascular risk. Cardio-oncology rehabilitation includes assessment of exercise capacity and individually prescribed aerobic and resistance exercise [49]. An international survey showed that rehabilitation is widely delivered during HSCT, using functional assessments such as the chair-rise test, grip strength and the 6 min walk test, with concurrent monitoring of blood counts [3].

### 4.4. Guidelines and Recommendations

Contemporary oncological rehabilitation is shifting from a reactive model towards prehabilitation and early mobilization. Physiotherapy should include functional assessment, education, exercise, respiratory and thromboprophylactic measures and fatigue management [50,51]. The World Health Organization (WHO) recommends rehabilitation at every stage of cancer care, focused on quality of life and independence [52]. ASCO (American Society of Clinical Oncology) guidelines recommend regular aerobic and resistance exercise during cancer treatment to reduce fatigue and preserve fitness and strength [53,54], and ASCO geriatric oncology guidelines recommend assessment of function, comorbidity and other vulnerabilities rather than reliance on chronological age [55,56]. In older patients with hematological malignancies, including those with multiple myeloma being considered for autoHSCT, actual fitness and physiological reserve should be assessed [57,58]. Mindfulness-based interventions, yoga, tai chi, cognitive-behavioral therapy, sleep hygiene and selected integrative medicine methods may support the management of anxiety, depression, fatigue and pain [59,60,61]. Care should be multidisciplinary and coordinated according to the individual needs of the patient [62,63].

## 5. Spa Rehabilitation and Balneotherapy: Current Evidence

### 5.1. Direct Evidence in Hematological Malignancies

No clinical studies evaluating balneotherapy or spa therapy in patients with hematological malignancies were identified, and no international guidelines address its use in this group. A systematic review of water therapies in cancer included 12 studies with 430 patients, conducted predominantly in patients with breast cancer, and none of the included studies focused on hematological malignancies [8]. Consequently, all statements in this review concerning spa-specific procedures are extrapolated (labels B–D).

It is, therefore, essential to distinguish between rehabilitation delivered in a spa setting and spa-specific procedures. The former relies on exercise-based interventions whose benefits in hematological malignancies are documented (Section 4) and which can be delivered in a spa as in any other setting [3,4,5,6]. The latter—balneotherapy, hydrotherapy, peloidotherapy and thermal and aerosol procedures—lack direct evidence of benefit, and their use should depend on procedure-specific qualification. Balneotherapy is not a systemic treatment and should not be equated with anticancer therapy. In patients with active disease, significant cytopenia, immunosuppression, infection or a high risk of bleeding, water-based procedures and other procedures involving environmental exposure require special caution or postponement [8,64,65,66].

### 5.2. Indirect Evidence from Oncology and Chronic Diseases

Hydrotherapy and aquatic exercise may improve pain, fatigue and quality of life in some breast cancer survivors, but the overall effectiveness of aquatic therapies remains equivocal, and the studies are heterogeneous and methodologically limited [8,67,68] (label C). A hydrotherapy-based supportive skin care program after breast cancer treatment has been evaluated in a randomized controlled study [14], and a multidisciplinary supportive and educational intervention delivered in a thermal resort has been evaluated after completion of breast cancer treatment [69]. In non-oncological chronic diseases, such as fibromyalgia and rheumatic diseases, balneotherapy has been associated with improvements in pain, function and quality of life [11,12,13] (label D). Evidence on physical therapy modalities in oncology is summarized in Section 8.

## 6. Biological Plausibility: Hypothesis-Generating Data Only

Balneotherapy and hydrotherapy can induce neuroendocrine and immunological responses that have been interpreted as anti-inflammatory, analgesic and adaptive (hormetic) effects [9,10,11]. In patients with osteoarthritis, fibromyalgia, chronic heart failure and cardiovascular risk factors, balneotherapy and peloidotherapy have been associated with reductions in selected pro-inflammatory cytokines, CRP (C-reactive protein), PGE2 (prostaglandin E2) and LTB4 (leukotriene B4), with changes in cortisol levels and innate immune responses, and with changes in blood pressure and selected cardiovascular markers [15,16,17,18,19,70,71,72,73,74,75].

Chronic inflammation influences hematopoietic stem cell function, clonal hematopoiesis and the development of myeloid malignancies, and its role is particularly evident in MPN Ph− [76,77,78,79]. This has prompted interest in whether the anti-inflammatory effects attributed to balneotherapy could be relevant to these diseases. However, all of the above data were obtained in non-hematological populations, mostly with short-term surrogate markers, and none demonstrates a clinical or disease-modifying effect in any hematological malignancy (label D). These data should be regarded as hypothesis-generating only and do not justify the use of spa-specific procedures in hematological patients for any purpose other than symptomatic and rehabilitative support.

## 7. Hydrotherapy, Peloidotherapy and Thermal and Aerosol Procedures

### 7.1. Thermal and Aerosol Procedures

Local application of heat may be considered for selected musculoskeletal complaints in clinically stable patients. Qualification should take into account thermal tolerance, skin condition, superficial sensation, hemodynamic stability and the location of possible neoplastic lesions. Sauna and other forms of whole-body thermotherapy require greater caution than local thermotherapy because of the load on the cardiovascular system and the risk of dehydration and impaired thermoregulation. In patients with fever, hemodynamic instability, significant dehydration, severe autonomic dysfunction or significant exercise intolerance, these procedures should be postponed. Whole-body cryotherapy is not documented in the rehabilitation of patients with hematological malignancies and should not be used routinely; there are insufficient data on its safety and effectiveness in this population [80,81,82] (label D).

Inhalation and aerosol therapy require consideration of the current infection risk, the way the procedure is performed and environmental conditions. In patients with active infection, fever, severe immunosuppression or severe neutropenia, group procedures should be postponed, and individual procedures should be preferred if the method is clinically justified.

There are no direct data on the safety and effectiveness of graduation towers (open-air brine evaporation structures used for inhalation of saline aerosol) in patients with hematological malignancies. Because of the possibility of exposure to aerosols and other environmental factors, special caution is warranted in patients with significant immunosuppression or severe neutropenia, and alternative, individual forms of rehabilitation should be considered. Risk assessment should take into account not only the type of substance or device used but also whether the procedure is carried out individually or in a group, and whether hygienic conditions and ventilation can be controlled (Table 3) [64,65].

**Table 3:** Thermal and aerosol procedures in patients with hematological malignancies: potential applications and safety aspects.

Procedure	Potential Use	Key Safety Aspects	Evidence Label
Local thermotherapy	Pain, muscle tension, selected musculoskeletal symptoms	Thermal tolerance, skin condition, sensation, hemodynamic stability	D
Sauna/whole-body thermotherapy	Relaxation, potential systemic effects	Circulatory load, dehydration and hemoconcentration (particularly in PV), thermoregulation, hypotension	D
Local cryotherapy	Pain, selected local symptoms	Neuropathy, circulatory disorders, skin condition, sensation	D
Whole-body cryotherapy	Potential symptomatic effect	Insufficient safety data; individual qualification	D (no data)
Individual inhalation	Selected respiratory indications	Infection, respiratory condition, procedure hygiene	D
Group aerosol therapy	Selected respiratory indications	Infectious exposure, ventilation, hygiene	D
Graduation towers	Potential supportive effect	Environmental and aerosol exposure, immunosuppression	D (no data)

Evidence labels as defined in Table 2; no clinical data in hematological malignancies were identified for any of these procedures. PV, polycythemia vera.

### 7.2. Hydrotherapy and Aquatic Exercise

Aquatic exercise can be part of the rehabilitation of patients with hematological malignancies, but its use requires consideration of the current clinical status, infection risk, skin condition and the presence of vascular access. In neutropenia, limiting exposure to potential sources of infection is particularly important, so individualized forms of rehabilitation conducted in a properly controlled environment may be preferred.

Infection risk increases steeply when the absolute neutrophil count (ANC) falls below 0.5 G/L (×10^9^/L), and this value defines severe neutropenia in infectious disease guidelines [83,84]. Guidelines for HSCT recipients recommend avoiding swimming in recreational water such as lakes and ponds and avoiding swallowing water during the period of immune recovery [66], and heated, aerosol-generating installations (whirlpools, hot tubs and spa pools) are a recognized source of *Legionella* and other waterborne infections [64,65]. However, no study has evaluated whether any ANC threshold predicts infection risk specifically during hydrotherapy, aquatic exercise or mineral baths. The ANC-based recommendations below are, therefore, extrapolated from general neutropenia and transplant infection-control data (label D).

On this basis, water-based procedures should be postponed in patients with ANC < 0.5 G/L or significant immunosuppression, during the early period after autoHSCT, alloHSCT or CAR-T therapy, and in patients with active infection or open wounds. The decision should consider not only the ANC but also fever or active infection, skin condition, vascular access, type of treatment and the sanitary conditions of the facility. An unhealed vascular access site, wound or skin damage are additional factors against the use of a swimming pool; in patients with a central venous catheter, eligibility for aquatic exercise should be discussed with the treating team on a case-by-case basis.

The risk associated with swimming pools and other water-based procedures depends primarily on the current immune status, blood counts, type of treatment and complications, and not solely on the time since remission. Great caution is warranted in active AML and during periods of intense immunosuppression; in stable remission of AML, controlled CML and MPN Ph−, access may be broader but always after individual clinical assessment. Whirlpool baths (hot tubs), intensively recirculating baths and other shared pools with a higher risk of microbiological exposure should be treated more restrictively than individual exercise in a controlled environment, and their use should be deferred in patients with significant immunosuppression, severe neutropenia, active infection, unhealed wounds or in the early post-HSCT period [64,65,66].

Restrictions on hydrotherapy should not be equated with a contraindication to rehabilitation itself. If water-based procedures are not recommended, rehabilitation should be continued, if the clinical condition allows, using individually selected methods that do not require water exposure (Table 4).

**Table 4:** Hydrotherapy and other water-based spa procedures in patients with hematological malignancies: potential benefits, risk factors and approach.

Procedure	Main Benefit	Main Risk	Approach in Hematological Malignancies	Evidence Label
Aquatic exercise	Functional training, weight relief	Infection, falls	May be considered individually	C (benefit); D (safety)
Shared (public) pool	Physical activity, hydrotherapy	Infectious exposure	Avoid in immunosuppression or ANC < 0.5 G/L; otherwise with caution	D
Whirlpool bath/hot tub	Relaxation, thermotherapy	Microbiological exposure (including *Legionella*), heat	More restrictive; defer in immunosuppression	D
Mineral bath (individual)	Hydro- and thermotherapeutic effect	Infection, hypotension, circulatory stress	Individual qualification	D

ANC, absolute neutrophil count; G/L, ×10^9^/L. Evidence labels as defined in Table 2.

### 7.3. Peloidotherapy

Peloidotherapy can be used symptomatically but should be considered an environmental and cutaneous exposure. In patients with hematological malignancies, it should be preceded by an assessment of the clinical condition, skin integrity, the presence of infection and the current immunological and hemorrhagic risk. In severe neutropenia, significant immunosuppression, active infection, skin damage or cutaneous chronic graft-versus-host disease (Section 9.2), the procedure should be postponed. In procedures involving significant thermal stress, heat tolerance, hydration and hemodynamic stability should also be considered [64,65] (label D).

The biological effects described in Section 6 should not be interpreted as evidence of antineoplastic activity or as a justification for using peloidotherapy to modify the course of a hematological malignancy. In the absence of clinical trials in this population, peloidotherapy should be regarded as a potential supportive intervention requiring individual qualification.

## 8. Physical Therapy Modalities in Hematological Malignancies

Physical therapy modalities are discussed because they are a routine part of spa rehabilitation programs (Section 3.1). Evidence for their use in oncology comes almost exclusively from other cancer populations, mainly breast and colorectal cancer and palliative care [85,86,87,88,89,90,91,92,93] (label C), and safety recommendations are derived from general cancer rehabilitation and electrophysical agent guidance [80,81,82,94] (label D).

Massage and selected manual therapy techniques can be used to reduce pain and muscle tension and to improve comfort. However, they require individual modification in thrombocytopenia, coagulation disorders, active bleeding, osteolytic lesions, venous thrombosis and skin damage. Direct pressure over active tumor sites, osteolytic lesions, wounds and vascular access sites should be avoided. The intensity and technique of massage should be adjusted to the current risk of bleeding and injury; in patients receiving anticoagulant or antiplatelet therapy, or Bruton tyrosine kinase (BTK) inhibitors, which increase bleeding risk, light-pressure techniques should be preferred [95,96]. In suspected or diagnosed acute thrombosis, massage of the affected area should be avoided. In osteolytic lesions, tumor involvement or suspected bone instability, deep manual techniques should be avoided in the affected areas [80,81,82,92,93].

TENS (transcutaneous electrical nerve stimulation) may be considered as an adjunctive treatment for pain, including selected neuropathic and musculoskeletal complaints. Electrodes should be placed outside the area of active or suspected cancer, and skin condition, superficial sensation and the presence of implanted electronic devices should be taken into account [82,89,90,91,94].

Photobiomodulation (PBM; low-level laser therapy, LLLT) may be useful for selected rehabilitation problems and symptoms associated with cancer treatment, such as lymphedema [87,88]. Because data on the effect of PBM on cancer tissue are inconclusive, routine use directly over active or suspected cancer should be avoided until more conclusive clinical data are available.

Therapeutic ultrasound may be used for selected musculoskeletal indications [85] but should not be applied directly over active or suspected cancer or over osteolytic lesions without prior clinical evaluation.

ESWT (extracorporeal shock wave therapy) may be considered for selected musculoskeletal problems and lymphedema in cancer patients [86] but should not be applied over active tumor sites, metastases or areas involved by multiple myeloma or lymphoma. Coagulation disorders, anticoagulation, thrombocytopenia and bone lesions that increase the risk of injury also require special attention. Qualification should consider the diagnosis, lesion location and current hematological status (Table 5).

**Table 5:** Selected physical therapy modalities in patients with hematological malignancies: potential applications and safety aspects.

Procedure	Potential Use	Key Safety Aspects	Evidence Label
Massage/manual therapy	Pain, muscle tension, comfort	Thrombocytopenia; anticoagulant, antiplatelet or BTK inhibitor therapy; bleeding; DVT; osteolytic lesions; wounds; vascular access sites	C (efficacy) [92,93]; D (safety)
TENS	Pain, selected neuropathic symptoms	Not over active or suspected cancer; skin condition and sensation; implanted electronic devices	C [89,90,91]; D (safety)
PBM/LLLT	Selected pain and soft tissue problems, lymphedema	Avoid routine use directly over active or suspected cancer	C [87,88]; D (safety)
Therapeutic ultrasound	Selected musculoskeletal problems, neuropathic symptoms	Not over active or suspected cancer or osteolytic lesions	C [85]; D (safety)
ESWT	Selected tendinopathies, lymphedema	Not over cancerous tissue or bone lesions; caution in thrombocytopenia and anticoagulation	C [86]; D (safety)

BTK, Bruton tyrosine kinase; DVT, deep vein thrombosis; ESWT, extracorporeal shock wave therapy; PBM/LLLT, photobiomodulation/low-level laser therapy; TENS, transcutaneous electrical nerve stimulation. Evidence labels as defined in Table 2.

## 9. Clinical Safety and Qualification Framework

### 9.1. Clinical Assessment

Assessment should include the stage of treatment, previous autoHSCT, alloHSCT or CAR-T therapy, current symptoms, infections, fever, dyspnea, bone pain, neuropathy, edema, skin condition, the risk of bleeding and fractures, medications and exercise tolerance. Hematological patients require simultaneous assessment of infectious, hemorrhagic, thrombotic, musculoskeletal and cardiovascular risk. In practice, exercise-based rehabilitation should be distinguished from spa-specific procedures: therapeutic exercise, functional training and education can be used more broadly, whereas hydrotherapy, peloidotherapy, swimming pools, inhalation and some physical therapy modalities require more stringent qualification [3,4,5,6,49,80].

### 9.2. Disease- and Treatment-Specific Considerations

Several situations require specific attention (Table 6). In PV, aquagenic pruritus is common—it was reported in 68% of 441 patients in one series and in 41% of 102 patients in a Polish cohort, one-third of whom avoided contact with water [97,98]—and may make baths and hydrotherapy poorly tolerated. Maintaining a hematocrit below 45% reduces the rate of cardiovascular death and major thrombosis [99]. Heat exposure (sauna, hot baths, whole-body thermotherapy) causes fluid loss and hemoconcentration; although not studied in PV, this mechanism argues for avoiding intense heat exposure, ensuring hydration and confirming hematocrit control before thermal procedures (label D).

After alloHSCT, chronic graft-versus-host disease (GVHD) frequently involves the skin, with sclerotic or lichen planus-like changes [100], and patients often receive prolonged immunosuppression, which increases infection risk. Fragile, sclerotic or photosensitive skin and ongoing immunosuppression argue against peloidotherapy, mineral baths and shared pools until the patient has been reviewed by the transplant team, and the infection-prevention recommendations for HSCT recipients apply [66] (label D).

After CAR-T therapy, cytopenias can be prolonged or biphasic (immune effector cell-associated hematotoxicity), and B-cell aplasia with hypogammaglobulinemia may persist for months or years, predisposing patients to infections [101,102]. Normal recent blood counts, therefore, do not exclude significant immunodeficiency; immunoglobulin levels, ongoing immunoglobulin replacement and infection history should be considered before water-based and aerosol procedures (label D). Immune effector cell-associated neurotoxicity syndrome (ICANS) should also be taken into account; it usually develops within the first weeks after infusion and may present with encephalopathy, aphasia, tremor, impaired handwriting, seizures or, rarely, cerebral edema [102]. Although ICANS is usually reversible, residual cognitive, motor or balance deficits may persist. Before spa rehabilitation, any history of ICANS and residual neurological deficits should, therefore, be documented; patients with such deficits require fall-risk assessment and supervision during water-based and thermal procedures, and any new neurological symptom should be treated as a STOP sign (Table 8) (label D).

Patients with multiple myeloma frequently have osteolytic lesions, osteoporosis and vertebral fractures and may have renal impairment and hypercalcemia [30,103]. Before manual therapy, massage, ESWT or weight-bearing and resistance exercise, a structured skeletal assessment should be performed, based on recent imaging and, where relevant, tools such as the Mirels score for long bones and the Spinal Instability Neoplastic Score (SINS), as recommended for exercise in people with bone metastases [104]. Renal function and calcium levels should be checked, and adequate hydration ensured, before thermal procedures (label D). Finally, patients receiving anticoagulants, antiplatelet agents or BTK inhibitors have an increased bleeding risk, which should be considered together with the platelet count when planning massage, manual therapy, ESWT and activities with a risk of falls [95,96] (label D).

**Table 6:** Disease- and treatment-specific safety considerations for spa rehabilitation in hematological malignancies.

Situation	Specific Risk	Practical Implication	Evidence Label
Polycythemia vera	Aquagenic pruritus; hemoconcentration and thrombosis with heat and dehydration	Assess water tolerance; avoid intense heat; ensure hydration; confirm hematocrit control (<45%) before thermal procedures [97,98,99]	B (pruritus); D (heat)
AlloHSCT with chronic GVHD	Skin fragility, sclerosis, photosensitivity; immunosuppression	Defer peloidotherapy, mineral baths and shared pools until reviewed by the transplant team; follow HSCT infection-prevention guidance [66,100]	D
After CAR-T therapy	Prolonged or biphasic cytopenias; B-cell aplasia, hypogammaglobulinemia; residual ICANS-related deficits	Check blood counts and immunoglobulin levels; consider infection history before water-based and aerosol procedures; document ICANS history and residual neurological deficits; assess fall risk [101,102]	D
Multiple myeloma	Osteolytic lesions, fractures; renal impairment, hypercalcemia	Structured skeletal assessment (imaging; Mirels score, SINS) before manual therapy, ESWT and loaded exercise; check renal function and calcium; ensure hydration [30,103,104]	D
Anticoagulant, antiplatelet or BTK inhibitor therapy	Increased bleeding risk	Light-pressure techniques; avoid deep massage and ESWT; fall prevention; interpret together with platelet count [95,96]	D

AlloHSCT, allogeneic hematopoietic stem cell transplantation; BTK, Bruton tyrosine kinase; CAR-T, chimeric antigen receptor T-cell; ESWT, extracorporeal shock wave therapy; GVHD, graft-versus-host disease; ICANS, immune effector cell-associated neurotoxicity syndrome; SINS, Spinal Instability Neoplastic Score. Evidence labels as defined in Table 2.

### 9.3. Hematological Parameters

Laboratory values should be interpreted together with symptoms, the trend of the values and the type of procedure, because hemoglobin (Hb), platelet count (PLT) and ANC can change rapidly; qualification should, therefore, be dynamic [3,4,5,6,7,49]. Table 7 presents the thresholds as a single ordered list, together with the source and origin of each value. Three points are essential for their interpretation.

First, there is no consensus on safe blood count values for exercise. A recent scoping review of 47 studies found that platelet thresholds were reported most often but varied widely, hemoglobin criteria (mostly <7–8 g/dL) were the most consistent, and neutrophil criteria were rarely numeric and usually triggered infection-control precautions rather than cessation of exercise [105]. The current trend is towards adapting rather than prohibiting exercise at low platelet counts [7,105], and the Academy of Acute Care Physical Therapy of the American Physical Therapy Association states that there is no absolute or relative platelet threshold for discontinuing physical therapy [106]. Second, all thresholds were derived from exercise and rehabilitation in hospital or outpatient settings (label B) or from general infection-control data (label D); none has been evaluated for spa-specific procedures. Third, the thresholds are pragmatic decision aids and should not be interpreted or applied as validated eligibility criteria for spa treatment.

**Table 7:** Laboratory parameters relevant to rehabilitation and spa-specific procedures in hematological malignancies, with the source and origin of each threshold.

Parameter	Suggested Approach to Exercise and Rehabilitation	Source and Origin of the Threshold (Label)	Spa-Specific Procedures
PLT < 10 G/L	Essential mobilization and ADL only; in some protocols exercise only after prophylactic transfusion; no resistance exercise; avoid falls	Prophylactic transfusion threshold; highest bleeding frequency in retrospective data; exercise trial protocols [7,105] (B)	Defer
PLT 10 to <20 G/L	Light aerobic and functional exercise under supervision; no resistance training; avoid falls and trauma; monitor blood pressure	Exercise trial protocols and safety studies [4,5,7,105] (B)	Defer procedures with trauma or fall risk; others by individual decision
PLT 20–50 G/L	Moderate intensity; light resistance (e.g., elastic bands); avoid vigorous and contact activities	Expert recommendations cited in reviews [7,80,105] (B/D)	Avoid deep massage and ESWT; other procedures with caution
PLT > 50 G/L	Progression, including loaded resistance training, if no other contraindications	Exercise trial protocols [105] (B)	No platelet-related restriction for most procedures
Hb < 7–8 g/dL	Pause or markedly reduce intensity; assess symptoms; transfusion decision by the hematologist	Most consistent criterion in exercise studies [105] (B)	Defer thermal and circulatory-stress procedures
Hb 8–10 g/dL	Symptom-guided intensity; Hb ≥ 10 g/dL is not an independent qualification criterion	Single studies and historical tables; no validated threshold [105] (B/D)	Caution with heat and hypotension
ANC < 0.5 G/L	Continue exercise individually with infection-control precautions (individual sessions, hygiene, avoidance of crowds)	Infection-risk gradient [83,84]; precautions used in exercise studies [105] (B/D)	Defer shared pools, hot tubs, group aerosol therapy and peloidotherapy
ANC 0.5–1.0 G/L	Individualized; hygiene precautions; consider fever, infection and trend	No exercise-specific evidence; authors’ consensus (D)	Individual decision; avoid shared hot tubs
Fever or active infection	No exercise beyond basic mobilization until medical assessment	Exclusion criterion in exercise studies [105]; infectious disease guidance [84] (B/D)	Postpone all spa-specific procedures

ADL, activities of daily living; ANC, absolute neutrophil count; ESWT, extracorporeal shock wave therapy; G/L, ×10^9^/L; Hb, hemoglobin; PLT, platelet count. Evidence labels as defined in Table 2. The thresholds are pragmatic considerations and not validated eligibility criteria for spa treatment.

### 9.4. Proposed Safety Framework

The STOP/MODIFY framework presented in Table 8 was developed by the authors based on the cancer rehabilitation and exercise safety literature; it has not been prospectively validated in spa rehabilitation. For each warning sign, Table 8 indicates whether it is adapted from existing studies or guidance or represents the authors’ consensus. In MODIFY situations, the session should be shortened, the intensity reduced, the number of breaks increased, and thermal or traumatic stimuli limited.

**Table 8:** Proposed STOP/MODIFY safety framework for rehabilitation and spa-specific procedures in hematological malignancies.

Warning Sign	Action	Basis (Label)
STOP: fever or suspected infection	Interrupt; medical assessment	Exclusion criterion in exercise studies [105]; cancer rehabilitation guidance [80] (B)
STOP: dyspnea at rest, chest pain, syncope or decreased oxygen saturation	Interrupt; urgent medical assessment	General exercise and cancer rehabilitation safety guidance [1,80] (D)
STOP: new neurological symptoms	Interrupt; medical assessment	Cancer rehabilitation safety guidance [80] (D)
STOP: significant bleeding	Interrupt; medical assessment	Exercise studies in thrombocytopenia [7,105] (B)
STOP: suspected DVT or PE	Interrupt; urgent medical assessment	Cancer-associated thrombosis guidance [95]; authors’ consensus (D)
STOP: acute bone or spinal pain, suspected fracture	Interrupt; imaging	Exercise consensus for bone metastases [104] (D)
MODIFY: increasing fatigue, decreased exercise tolerance	Shorten sessions, reduce intensity, increase breaks	Authors’ consensus based on [3,4,5,6,7,49] (D)
MODIFY: dizziness, balance disorders, orthostatic symptoms	As above; fall prevention; limit thermal stimuli	Authors’ consensus (D)
MODIFY: worsening neuropathy, tendency to bruising or hematoma	As above; limit traumatic and thermal stimuli; check blood counts	Authors’ consensus (D)

DVT, deep vein thrombosis; PE, pulmonary embolism. Evidence labels as defined in Table 2.

## 10. Polish Regulatory Framework and Proposed Qualification Pathway

### 10.1. Current Polish Regulations

The considerations in this section apply specifically to the Polish health system; eligibility rules for spa treatment differ between countries, and readers from other systems should interpret this section accordingly. In Poland, referral to and qualification for spa treatment and spa rehabilitation financed by the National Health Fund are governed by regulations of the Minister of Health [107,108]. Among the general contraindications, the regulation lists active neoplastic disease and a period shorter than 5 years (malignant melanoma, leukemia, Hodgkin lymphoma, malignant lymphomas and kidney cancer) or shorter than 12 months (other malignant neoplasms) since the completion of surgical treatment, chemotherapy or radiotherapy, excluding hormonal therapy [107].

This approach ensures uniform and prudent formal criteria, but the time since completion of treatment does not necessarily reflect the patient’s current clinical condition or actual need for rehabilitation. Some patients experience sequelae of the disease and its treatment for many years, such as chronic fatigue, limited physical capacity, muscle weakness, neuropathy, pain or reduced quality of life, whereas others remain clinically stable and would meet individual safety criteria for rehabilitation well before the end of the waiting period. In addition, in patients with chronic diseases receiving continuous targeted therapy (e.g., CML or CLL), the point of “completion of treatment” may be difficult to define (the proposed approach for these patients is described in Section 10.2).

### 10.2. Proposed Early Qualification Pathway

**Figure 1:**
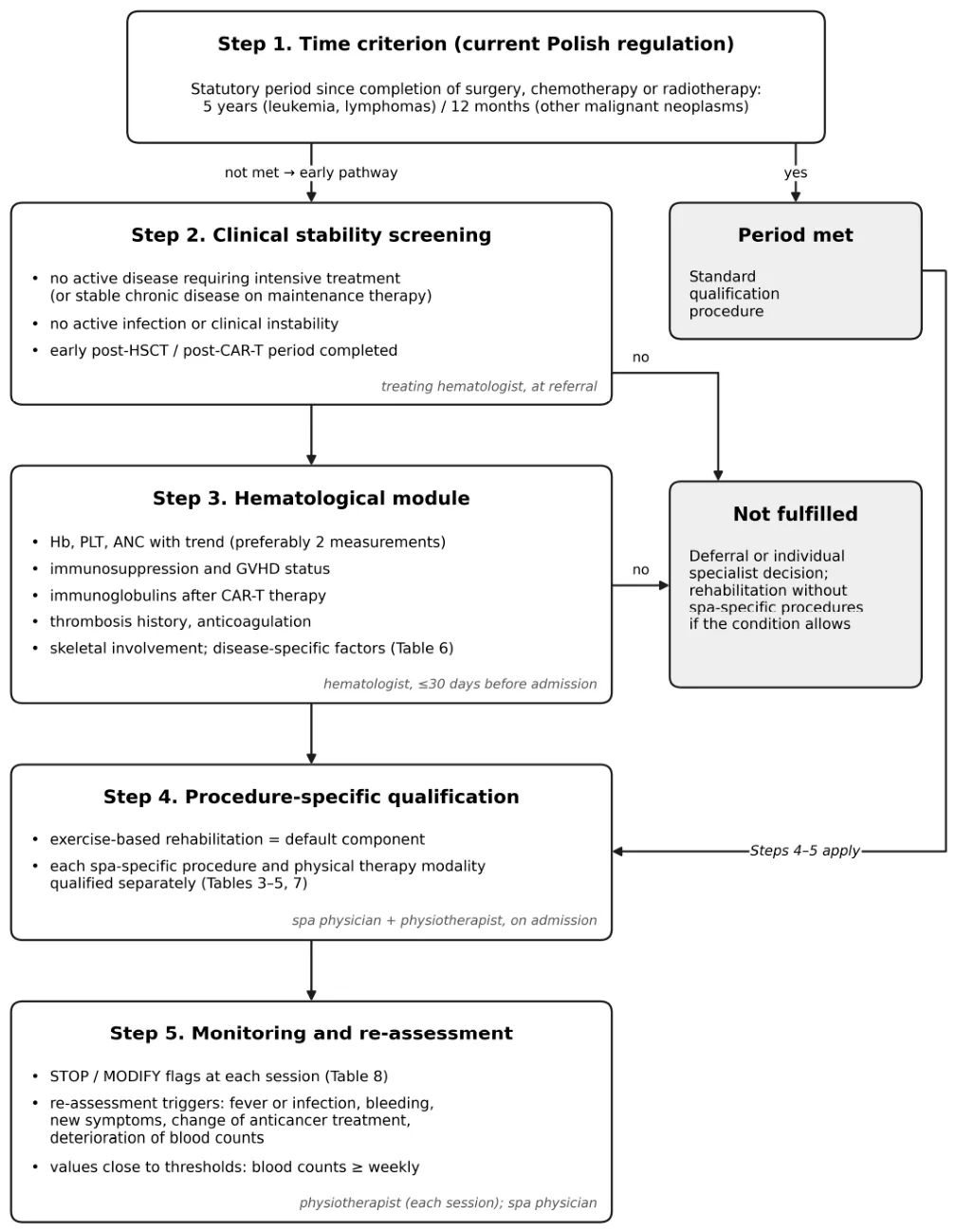
Proposed early qualification pathway for spa rehabilitation in patients with hematological malignancies (authors’ proposal; not validated). The pathway supplements, rather than replaces, the statutory time criterion. Patients receiving continuous therapy without a defined end date (e.g., CML, CLL) enter the early pathway directly at Step 2. ANC, absolute neutrophil count; CAR-T, chimeric antigen receptor T-cell therapy; GVHD, graft-versus-host disease; Hb, hemoglobin; HSCT, hematopoietic stem cell transplantation; PLT, platelet count.

As a hypothesis for prospective evaluation, we propose an early qualification pathway in which the time criterion is supplemented, not replaced, by a structured clinical assessment (Figure 1). The pathway would not automatically admit patients before the end of the statutory period; it would allow selected patients to be qualified earlier if an objective assessment confirms the absence of active disease requiring intensive treatment, adequate clinical stability and the feasibility of safe rehabilitation. Patients with active disease, clinical instability, significant immunosuppression, active infection, a significant risk of bleeding or other significant contraindications would remain subject to deferral or individual specialist decision making.

The pathway consists of five steps. (1) Time criterion: patients who meet the current statutory period follow the standard procedure, whereas those who do not may enter the early pathway. We propose that patients receiving continuous therapy without a defined end date (e.g., tyrosine kinase inhibitors in CML or continuous BTK inhibitor therapy in CLL), who by definition cannot meet the time criterion, enter the early pathway directly, with eligibility determined by disease stability on therapy in Step 2; in patients who have completed fixed-duration therapy (e.g., venetoclax-based regimens in CLL) or have discontinued therapy (e.g., treatment-free remission in CML), the period would be counted from the date of the last dose. (2) Clinical stability screening, performed by the treating hematologist at referral: no active disease requiring intensive treatment (or stable chronic disease on continuous or maintenance therapy), no active infection, no clinical instability and completion of the early post-HSCT or post-CAR-T period, as defined by the treating center. (3) Hematological module, completed by the hematologist no earlier than 30 days before admission: Hb, PLT and ANC with their trend (preferably two measurements), immunosuppression and GVHD status, immunoglobulin levels and ICANS history after CAR-T therapy, thrombotic history and anticoagulation, skeletal involvement and the disease-specific factors listed in Table 6. (4) Procedure-specific qualification, performed on admission by the spa physician together with the physiotherapist: exercise-based rehabilitation is the default component, and each spa-specific procedure (balneological, thermal or aerosol) and physical therapy modality is qualified separately according to Table 3, Table 4, Table 5 and Table 7. (5) Monitoring and re-assessment during the stay: red flags (STOP) or yellow flags (MODIFY) (Table 8) trigger interruption or modification of the program; re-assessment by the spa physician, with consultation of the treating hematologist when needed, is required in the event of fever or infection, bleeding, new symptoms, a change in anticancer treatment or deterioration of blood counts, and in patients qualified with values close to the thresholds in Table 7, blood counts should be repeated at least weekly.

### 10.3. Requirements for Implementation

The proposed pathway has not been evaluated in terms of safety, effectiveness, resource use or impact on the health system, and its potential benefits—shorter delays between the end of treatment and the start of rehabilitation and a lower risk of persistent functional deficits—remain hypothetical. Its implementation would require amendment of the relevant regulations and the development and validation of uniform clinical criteria, so that the decision on early qualification does not depend solely on the discretionary judgment of a single physician. The pathway should first be evaluated in a pilot program in terms of safety, functional outcomes, quality of life, costs and waiting times (Section 12). In principle, the time criterion should not replace clinical judgment but should be integrated with it, so that the possibility of starting rehabilitation earlier depends on the patient’s current, objectively assessed risk profile.

## 11. Limitations

This review has several limitations. First, it is a narrative rather than a systematic review; although the search strategy and selection process are reported, incomplete retrieval of relevant studies cannot be excluded. The search was restricted to publications in English and Polish. Second, no formal risk-of-bias or certainty assessment of individual studies was performed; the evidence labels used (Table 2) describe the directness of the evidence, not its internal validity. Third, no direct evidence on spa-specific procedures in hematological malignancies was found, so all recommendations concerning these procedures are extrapolated from other populations or represent the authors’ consensus. Fourth, the laboratory thresholds and the STOP/MODIFY framework have not been validated for spa-specific procedures in any setting. Fifth, the regulatory analysis and the proposed qualification pathway refer to a single country (Poland) and may not be transferable to other health systems. Finally, some authors are professionally connected with spa treatment and with the organization that funded this work (see Conflicts of Interest); although the conclusions were formulated conservatively, this should be taken into account when interpreting the proposed change in qualification rules.

## 12. Evidence Gaps and Future Research

There are no guidelines from hematological societies on the use of spa-specific procedures or physical therapy modalities in leukemia, lymphoma and myeloma. Most data come from general oncology and require adaptation to the specific characteristics of hematological patients, particularly cytopenias, bleeding risk and infection risk, and there are no large randomized controlled trials assessing the safety and efficacy of physical therapy modalities in patients with active hematological malignancies.

We suggest the following steps: First, a prospective registry of patients with hematological malignancies admitted to spa rehabilitation in Poland, recording the diagnosis, treatment history (including HSCT and CAR-T therapy), time since treatment, blood counts, procedures received and adverse events. Second, a prospective pilot safety and feasibility study of the early qualification pathway with predefined stopping rules, in which the primary endpoints should include adverse events graded according to the Common Terminology Criteria for Adverse Events (CTCAE v5.0), clinically and microbiologically documented infections, bleeding events graded on the WHO bleeding scale and falls, together with feasibility measures (recruitment, retention and adherence); secondary endpoints should include the 6 min walk test, fatigue (FACIT-Fatigue), quality of life (EORTC QLQ-C30) and the time from completion of treatment to the start of rehabilitation. Third, randomized comparisons of exercise-based rehabilitation with and without spa-specific procedures, to determine whether these procedures add any benefit beyond exercise. Fourth, environmental monitoring of pools and aerosol-generating installations (e.g., for *Legionella* and *Pseudomonas*) used by immunocompromised patients.

## 13. Conclusions

In patients with hematological malignancies, the documented benefits concern exercise-based and functional rehabilitation, which improves physical capacity, fatigue and quality of life and can be delivered safely in selected patients, including those with cytopenias, under appropriate supervision [3,4,5,6,7,105]. Such rehabilitation can be delivered in a spa setting, but there is no direct evidence that the spa setting itself or spa-specific procedures, such as balneotherapy, hydrotherapy and peloidotherapy, provide additional benefit in this population. These procedures should be regarded as optional adjuncts requiring procedure-specific qualification because of their infectious, hemorrhagic, thermal and skin-related risks.

Qualification should be based on the current clinical and functional status, disease- and treatment-specific factors and blood counts, with the program developed jointly by a hematologist, a rehabilitation or spa physician and a physiotherapist. The laboratory thresholds and the safety framework presented here are pragmatic, largely extrapolated and require prospective validation. The proposed earlier qualification pathway within the Polish system is a hypothesis; its introduction should be preceded by the development and validation of uniform criteria and by an evaluation of safety, functional outcomes, quality of life, resource use and waiting times.

## Figures and Tables

**Table 1:** Number of new cases of selected malignant neoplasms of lymphoid and hematopoietic tissue in Poland in 2023, according to the Polish National Cancer Registry [42].

ICD-10 Code	Diagnosis	Men	Women	Total
C91	Lymphoid leukemia	1472	1143	2615
C83	Non-follicular lymphoma	1170	1038	2208
C90	Multiple myeloma and malignant plasma cell neoplasms	1084	1035	2119

**Table 2:** Evidence-certainty labels used in this review.

Label	Definition	Example
A	Direct evidence: studies in patients with hematological malignancies evaluating the specific intervention or setting	Exercise during and after HSCT
B	Direct population, extrapolated intervention: studies in hematological malignancies evaluating related (land-based) exercise or rehabilitation, applied to spa-specific procedures	Platelet thresholds for exercise applied to spa programs
C	Indirect population: studies in other oncological populations	Aquatic exercise in breast cancer survivors
D	Mechanistic data, studies in non-oncological populations, general guidance (e.g., infection control) or authors’ consensus; includes absence of data	Restriction of hot tubs in severe neutropenia

HSCT, hematopoietic stem cell transplantation.

## Data Availability

No new data were created or analyzed in this study. Data sharing is not applicable to this article.
